# Immune Dysfunction in Medication-Related Osteonecrosis of the Jaw

**DOI:** 10.3390/ijms24097948

**Published:** 2023-04-27

**Authors:** Ilaria Roato, Rodolfo Mauceri, Vincenzo Notaro, Tullio Genova, Vittorio Fusco, Federico Mussano

**Affiliations:** 1 CIR-Dental School, Department of Surgical Sciences, University of Turin, 10126 Turin, Italy; vincenzo.notaro@unito.it (V.N.); federico.mussano@unito.it (F.M.); 2 Department of Surgical, Oncological and Oral Sciences, University of Palermo, 90133 Palermo, Italy; rodolfo.mauceri@unipa.it; 3 Department of Life Sciences and Systems Biology, University of Torino, 10123 Torino, Italy; 4 Medical Oncology Unit, Azienda Ospedaliera SS. Antonio e Biagio e Cesare Arrigo, 15121 Alessandria, Italy; 5 Department of Integrated Research Activity and Innovation (DAIRI), Azienda Ospedaliera SS. Antonio e Biagio e Cesare Arrigo, 15121 Alessandria, Italy

**Keywords:** medication-related osteonecrosis of the jaw, osteoclast, osteoblast, antiresorptive drug, immune system

## Abstract

The pathogenesis of medication-related osteonecrosis of the jaw (MRONJ) is multifactorial and there is a substantial consensus on the role of antiresorptive drugs (ARDs), including bisphosphonates (BPs) and denosumab (Dmab), as one of the main determinants. The time exposure, cumulative dose and administration intensity of these drugs are critical parameters to be considered in the treatment of patients, as cancer patients show the highest incidence of MRONJ. BPs and Dmab have distinct mechanisms of action on bone, but they also exert different effects on immune subsets which interact with bone cells, thus contributing to the onset of MRONJ. Here, we summarized the main effects of ARDs on the different immune cell subsets, which consequently affect bone cells, particularly osteoclasts and osteoblasts. Data from animal models and MRONJ patients showed a deep interference of ARDs in modulating immune cells, even though a large part of the literature concerns the effects of BPs and there is a lack of data on Dmab, demonstrating the need to further studies.

## 1. Introduction

Osteonecrosis of the jaw (ONJ) is a multifaceted disease that has been known since 2003 in patients exposed to BPs (bisphosphonate-related ONJ (BRONJ)), but was renamed medication-related ONJ (MRONJ) [1] after the observation of cases due to the receptor activator of the nuclear factor κB ligand (RANKL) antibody, denosumab (Dmab), and other drugs [2,3,4]. Indeed, due to the continuous evolution in cancer treatment, MRONJ has been associated in smaller measure to antiangiogenic and cyclin inhibitor drugs [2,5].

To date, there is still open discussion about the definition, diagnosis, staging and treatment strategy of MRONJ [5,6,7,8,9,10]. The pathogenesis of MRONJ appears multifactorial [11]. MRONJ has the peculiarity of occurring in the jaw (often, albeit not exclusively, at a tooth extraction site), but not in other bones. A possible reason is the strong mechanical stimulation to which jaws are subjected that leads to a high bone turnover rate, which is approximately 3- to 6-times faster than those observed in long bones of beagle dogs [12,13].

There is a substantial consensus on the important role of antiresorptive drugs (ARDs), such as BPs and Dmab, in the pathogenesis of MRONJ [1,4,14]. With regard to zoledronic acid (Zol), which is an amino-bisphosphonate (N-BP), and denosumab (Dmab), time exposure, cumulative dose and administration intensity are all parameters that increase MRONJ risk. Of course, MRONJ reduces the quality of life of the affected patients [15]; thus, measures aimed to reduce the disease risk that contribute to patients’ oral health have been adopted by clinicians.

The coexistence of more factors increases the risk for MRONJ, which is higher for oncologic than osteoporotic patients likely due to the higher and more frequent doses of ARDs that can lead to an intense suppression of bone turnover [16]. One of the main causes of MRONJ is represented by the inhibition of osteoclast (OC) and osteoblast (OB) activity due to the ARDs, which causes suppressed bone turnover with compromised bone healing [17]. Microcirculation dysfunctions with angiogenesis inhibition [18], mucosal damage secondary to toxic exposure of the bone, bacterial infection and immune dysfunction all come together to lead to MRONJ [17,19].

Here, we will focus on the alterations of different immune aspects leading to MRONJ that are caused by the two most used ARDs: Zol, an amino-bisphosphonate (N-BP), and Dmab, the receptor activator of the nuclear factor κB ligand (RANKL) antibody.

## 2. An Immunosuppressed Milieu Favors ONJ Induced by ARDs

Both N-BPs and Dmab cause an immune dysfunction in MRONJ patients [20,21,22] by hindering their capability to respond properly to immunological stress independently of the oral microbiome [23]. It is also noteworthy that a large number of patients that develop MRONJ have other disease conditions or partake in many pharmacological treatments (chemotherapy, steroids, antiviral drugs, etc.), which may contribute to their immune system impairment [23,24].

The role of immune responses and inflammation in the onset and/or progression of MRONJ has been recently reported since a massive infiltration of lymphocytes mixed with inflammatory cells within tissue affected by MRONJ has been documented [25]. Moreover, it has been found that N-BPs increased the production of acute general inflammatory mediators in vitro [26] and in vivo [27], modifying the immune cell subset of patients [28,29], but they did not change inflammatory bone markers. Tooth extraction is comparable, for some aspects, to a bone fracture, where inflammation and fracture healing are parallel processes, because both need a focus and a resolution. Initially, at the damage site, the T-cell subset releases cytokines, such as IL-17, which directly support the proliferation and differentiation of local mesenchymal stem cells into OBs. Later, another specific subgroup of T cells blocks the secretion of pro-inflammatory factors to allow for lesion healing. In pathological conditions, the over production of IL-17 elicits an opposite effect on OBs by inhibiting their differentiation and activity, and by promoting OC bone resorption [30,31]. Thus, the correct cross-talk among immune cells and bone cells is fundamental to avoid both bone and immune alterations [32]. In mice treated with Zol, tooth extraction increases inflammatory cytokine levels and osteocyte apoptosis in the extraction site, promoting osteonecrosis [33]. These data confirm other previously published data, showing that the serum level of inflammatory cytokines was increased in MRONJ patients and that the administration of anti-inflammatory cytokines, such as antitumor necrosis factor-α (TNFα) and anti-interleukin 6 (IL-6), were effective in preventing a cytokine storm induced by N-BPs [33]. Recently, it has been reported in a murine model that the administration of either anti-inflammatory or antibiotic drugs significantly blocked Zol-induced osteonecrosis following tooth extraction [34], suggesting that this type of treatment should be considered to prevent MRONJ onset.

## 3. Bacterial Infections as Both Cause and Consequence of Immune Dysfunction in MRONJ

The role of commensal oral microbiota and bacterial infections, either associated or not to tissue damage induced by invasive dental procedures, in MRONJ is debated [35]. It is noteworthy that the jawbone is the peculiarly susceptible to infections compared to other bones, which are not as easily exposed to microorganisms as they occur in the oral cavity. Breaching the mucosal barrier during or after antiresorptive treatment may cause infection and hinder the healing process, thus leading to bone necrosis [36,37]. The most common surgical procedure associated with the onset of osteonecrosis is tooth extraction [6]. After N-BP treatment, bacteria are known to stimulate bone resorption [38,39]. A growing number of scientific papers have suggested the possible role of Actinomyces species [40,41], which are ubiquitous Gram-positive, non-spore-forming bacteria, that were found in more than 80% of bone samples from MRONJ patients in two retrospective studies [37,42]. Bacterial microfilms that are detectable in N-BP-related sites of osteonecrosis may stimulate OC activity on the bone surface, supporting the concept that microorganisms may directly contribute to bone necrosis [43,44].

Several studies highlighted oral cavity infection as a major event that stimulates a chronic inflammatory immune response, with the increase in cytokines leading to the upregulation of β-defensin 3 [45]. Defensins are antimicrobial peptides (AMPs) that are important in the innate immunity response against microbial pathogens [46], and they exert a protective action on oral cavity integrity against the invasion by microbes [47]. In the animal model, it has been reported that infectious osteomyelitis and ARD administration synergize in promoting MRONJ, with an increased release of pro-inflammatory cytokines. The same authors suggested that ”pro-inflammatory cytokines may represent therapeutic targets to prevent osteonecrosis induced by infectious osteomyelitis in patients treated with anti-resorptive therapy” [48,49]. Increased levels of β-defensins were also described in osteomyelitis of the jaw compared to uninfected healthy jaws, while in infected osteoradionecrosis (ORN), their levels were significantly reduced [50]. This observation suggests that, in MRONJ, bone displays not only necrotic characteristics similar to the ORN samples, but it shows the previously described aspect of bone affected by bacterial infections [51]. Thus, Stockman et al. concluded that “The increased expression of human β-defensins in bone samples of N-BP-induced ONJ can be interpreted as a sign of unimpaired metabolic activity and can therefore be seen as a reaction of vital bone to microbial invasion” [50]. Furthermore, β-defensins are expressed by OBs, stimulating their proliferation and differentiation process [52], and, in patients with infection, the level of expression of β-defensin-2 by OBs has been found to increase, suggesting that antimicrobial peptides play a central role in the prevention of bone infection. Looking at patients treated with immunosuppressive drugs, an increased susceptibility to bone infection seems to occur due to decreasing antimicrobial peptide expression levels [53]. Considering all these data and the fact that an intrinsic basal level of β-defensin 3 expression is independent of exposure to bacterial stimuli, it is still unclear whether AMP expression contributes to the MRONJ pathogenesis or if it is simply an after-effect of the disease [54].

## 4. Antiresorptive Treatments Exert Different Effects on Immune System Subsets

The role of different immune cell subsets, such as γδ T cells [21], T helper 17 (Th17) and T regulatory (T reg) cells [55], natural killer (NK) cells [56], dendritic cells (DCs) [57], neutrophils [58] and macrophages [55,59], has been investigated in the pathogenesis of MRONJ. Based on the scientific literature, it appears evident that all of these immune cell subsets concur with ONJ onset induced by Dmab and Zol, but a core function in the pathogenesis seems to be played by myeloid immune cells. Myeloid cells are recruited in injured barrier tissues where they orchestrate wound healing. Zol has been demonstrated to interfere with this healing process, directly affecting myeloid cells. Indeed, in a mice model, the administration of Zol followed by tooth extraction caused osteonecrotic lesions, which was dependent on an increased localization of Ly6G+/Gr1+ myeloid cells in the oral barrier. These cells showed abnormal morphology and size. The administration of an antibody anti-Ly6G caused the depletion of these myeloid cells with a reduction in osteonecrotic lesions [60]. This result suggests that the local modulation of myeloid cells in oral barrier tissue is a fundamental step in the pathogenesis of MRONJ.

**Figure 1:**
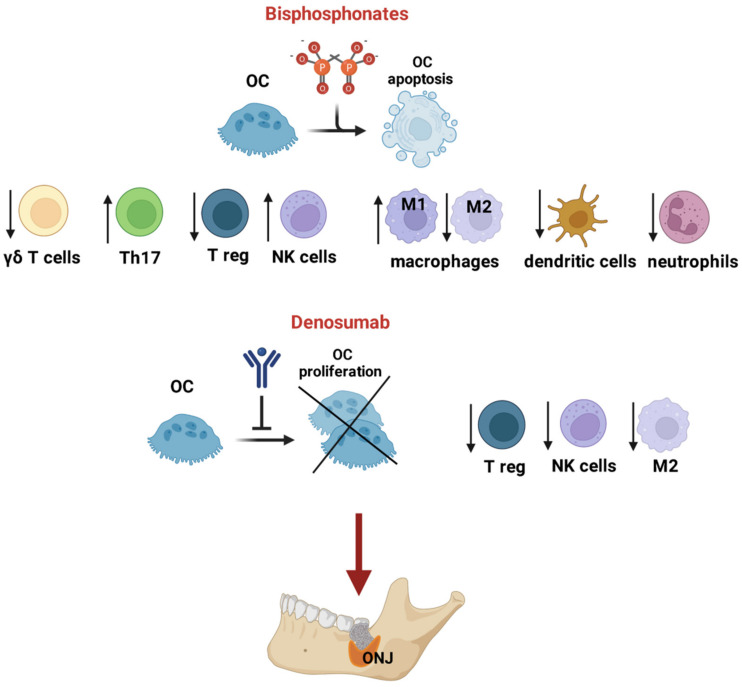
Effects of BPs and Dmab on OCs and immune cells that possibly cause MRONJ. BPs act on OCs, inducing their apoptosis, and exert an inhibitory effect on γδ T cells, regulatory T cells (Tregs), dendritic cells and neutrophils. BPs stimulate Th17 and affect the balance of macrophages, reducing the pro-inflammatory M1 and inducing the phenotype M2. Dmab links RANKL by inhibiting OC proliferation and activation, while its reported effect on immune system subsets is limited to a reduction in the anti-inflammatory macrophage M2. At last, the effect of both BPs and Dmab contribute to the onset of ONJ. Created by Biorender (accessed on 28 February 2023).

A huge effort has been made to unravel the effect of N-BPs on the modulation of different immune subsets, while only a few studies have been conducted regarding the action of Dmab on immune cells. It is likely that this lack of knowledge on Dmab depends on the fact that Dmab can be studied only in human and not in mice models. Notably, different research studies have been conducted using RANK-Fc or OPG-Fc, which mimic Dmab action in mice, but they are not the molecule for human use. The picture that emerges from the literature allows us to understand that BPs demonstrate immunosuppressive activity on all of the main immune subsets, while for Dmab, it has been reported to carry out modulatory activity on anti-inflammatory macrophages, as well as on T-reg and NK cells (Figure 1).

### 4.1. γδ T Cells

γδ T cells represent 1% to 10% of nucleated cells in the human peripheral blood, while their number is higher in tissues, particularly in epithelia [61]. γδ T cells are innate immune cells and are a kind of common immune barrier effector cell; their T-cell receptor (TCR) specificity is directed almost exclusively toward non-peptide antigens [62]. They play a role in homeostasis, adaptation to stress and wound healing [63,64] in addition to having anticancer and antiviral capabilities [65,66]. A gene expression analysis conducted on leukocytes of patients who had experienced N-BP-related ONJ and were deficient in γδ T cells showed a low expression of factors important for immunity and wound healing, such as the receptor activator of NF-kB (RANK), RANKL, TNFα, granulocyte-macrophage colony-stimulating factor (GM-CSF), connective tissue growth factor (CTGF) and matrix metalloproteinase-7 (MMP7) [67].

Among the inflammatory cytokines, interferon γ (IFNγ) is highly expressed by γδ T cells, and it is known to play a key role in the early defensive immune response to pathogenic microorganisms [68] and in the regulation of osteoclastogenesis [69]. Thus, one hypothesis is that γδ T cells may produce IFN-γ, which suppresses osteoclastogenesis by interfering with the RANKL-RANK signaling pathway and promoting jaw necrosis. After a first infusion of N-BPs, patients often develop an acute-phase reaction due to the activation of γδ T cells and the consequent release of cytokines such as IFNγ [70]. Interestingly, after repeated administrations of N-BPs, a decline in circulating γδ T cells was reported in [28]. Indeed, an observational clinical study on osteoporotic patients treated with N-BPs showed a significant deficiency in Vγ9Vδ2 T cells after 1 year of treatment with oral or intravenous N-BPs [24]. This N-BP-related deficiency in circulating γδ T cells was directly correlated to the length of time of N-BP exposure and to the administration route [24]. γδ T cells act in the early phase of the healing process by releasing IL-17, which must be tightly regulated both spatially and temporally to allow tissue regeneration [32]. IL-17, which is normally produced by γδ T cells present in the injured site, supports mesenchymal stem cell proliferation and differentiation into OBs [71]. When IL-17 is largely released by Th17 cells, such as in pathological conditions such as rheumatoid arthritis, it can negatively affect already mature OBs and osteocytes, stimulating OCs at the same time and causing consequent bone loss [31,72]. In the mucosal tissue that is close to the non-healing extraction sockets of MRONJ patients, a significant increase in Th17 cells and IL-17 was reported in [55], whereas Treg cells were found to be reduced, contributing to the prolonged inflammatory state.

Looking at the γδ T cells present in the oral mucosal barrier, both animal models deficient in γδ T cells, such as γδ-T-cell null mice and wild-type controls, treated with Zol showed histological signs of osteonecrosis, whereas osteonecrosis was not induced when injecting human γδ T cells into Zol-treated immunodeficient (Rag2^−/−^) mice [21]. This suggests that γδ T cells do not play a central role in the osteonecrosis mechanism, but rather they contribute to the variability in the different oral mucosal diseases associated with MRONJ [21]. Conversely, an interesting work by Movila et al. reported on a crucial role of γδ T cells in the pathogenesis of MRONJ [73]. The authors showed that activated γδ T cells produced semaphorin-4D (Sema4D) in vitro, which stimulates TNFα production from macrophages. γδ-T-cell KO mice were resistant to ONJ induction and they did not show the local production of Sema4D and TNF-α. Moreover, the systemic administration of anti-Sema4D neutralizing mAb suppressed the onset of osteonecrosis in wild-type mice, and the levels of TNFα were reduced [74]. Thus, an association between γδ T cells and osteonecrosis exists, but a direct cause–effect relationship is not clear.

N-BPs cause the accumulation of a metabolite, isopentenyl pyrophosphate (IPP), in the mevalonate pathway of the cholesterol metabolism [75,76,77], which is an antigen identified by γδ T cells that initially stimulates them, but later induces their reduction through a mechanism of activation-induced cell death, commonly used by the immune system to maintain homeostasis [28,78,79]. Another hypothesis explains the loss of γδ T cells with the high release of reactive oxygen species from neutrophils taking up N-BPs, which results in γδ-T-cell suppression [58]. Although data on γδ T cells in Dmab-treated patients are few, it appears relevant to understand whether and how γδ T cells play a role in Dmab-induced ONJ. Dmab does not interfere with the mevalonate pathway because it is a human anti-RANKL antibody capable of blocking RANK/RANKL signaling, affecting OC bone resorption and, at the same time, regulating the immune system. Indeed, RANKL is expressed by activated T cells; it is necessary for thymic selection during the development of γδ-T-cell progenitors [80], and it controls the DC function [81]. Thus, Kalyan hypothesized that Dmab lowers the immune defenses by reducing T-cell development [23]. All these data suggest that γδ T cells contribute to ONJ pathogenesis, but they do not mediate the core mechanism for ONJ onset; this was also demonstrated by Park et al. [21], who observed Zol-induced ONJ in both wild-type and γδ-T-cell null mice.

### 4.2. T Regulatory Cells and T Helper-17 Cells

T reg cells are a subset of CD4+CD25+ T cells expressing CD4, CD25 and FOXP3 that play a critical immunoregulatory role, contributing to the immunosuppressive response [82,83]. In a mice model, Zol was shown to induce ONJ by modulating the Treg/Th17 ratio. In particular, Tregs were suppressed, while Th17 cells were activated, promoting inflammation [55]. The reintroduction of Tregs in these mice restored the Treg/Th17 ratio and prevented ONJ after extraction [84].

RANKL induces the generation of Tregs; hence, Dmab affects Treg response and their suppressive function [85]. RANKL initially stimulates the immune response, increasing the survival of DCs with their antigen presentation activity and causing T-lymphocyte trafficking [86]. Subsequently, RANKL induces a Treg-mediated response [82,83], restraining inflammation and bone loss. To demonstrate a cause-and-effect relationship between the Treg and RANKL immunoregulatory effects, Francisconi et al. used a murine model of a periapical lesion, in which treatment with an anti-RANKL reduced bone damage, but did not interfere with the immuno-inflammatory characteristics of the lesion [20]. By maintaining a pro-inflammatory microenvironment, RANKL inhibitors promote MRONJ in mice with periapical lesions, as was also previously shown by other groups [87,88]. The adoptive transfer of Tregs in these mice prevented lesion relapse after anti-RANKL treatment discontinuation [20]. In other studies, RANKL promoted Treg generation in thymic and tumor microenvironments, and its inhibition caused an increase in effector T-cell responses [89,90]. It is therefore possible that Dmab exerts a direct effect on Tregs as well as on RANK-Fc (a recombinant RANKL antagonist) in MRONJ, although further research is required.

### 4.3. Natural Killer Cells

Natural killer (NK) cells are effector cells that exert a direct natural cytotoxicity and antibody-dependent cellular cytotoxicity (ADCC). NK cells also produce many cytokines and chemokines, indirectly affecting different immune cellular subsets [60,91].

**Figure 2:**
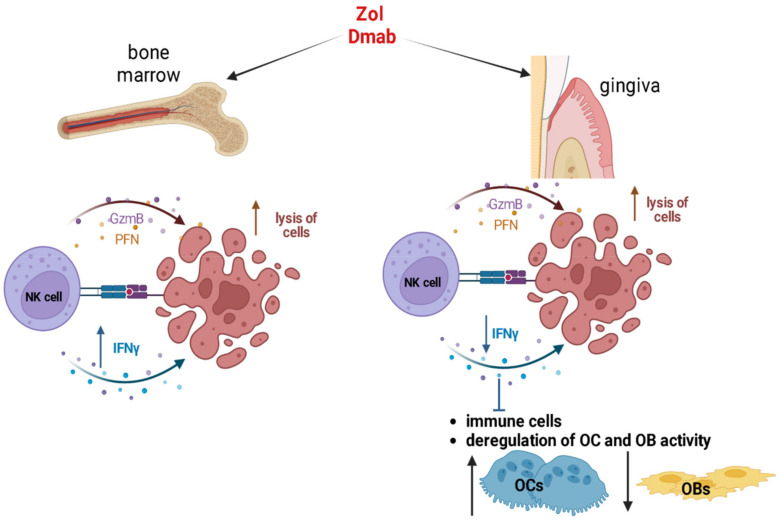
Effects of Zol and Dmab on NK cells. In bone marrow, Zol and Dmab exert a stimulatory effect on NK cells, increasing IFNγ release and cytotoxic activity mediated by the release of granzyme (GzmB) and perforin (PFN), which cause cell killing. In gingiva, Zol and Dmab stimulate NK cell cytotoxic activity and inhibit IFNγ secretion, which results in an inhibition of immune cells and the unbalanced activity of OCs and OBs. Created by Biorender (accessed on 28 February 2023).

In a humanized-BLT mouse model, Kaur et al. investigated how Zol and Dmab modulate NK cell activity, showing a different effect depending on the studied tissues. Indeed, these ARDs increased NK cells’ cytotoxic activity and IFNγ release in bone marrow, while in gingiva they reduced the secretion of IFNγ, even though the NK cytotoxicity was high (Figure 2). NK cells present in gingiva represent a further inhibitory immune subset in addition to T cells, as they are locally primed and partially activated owing to the strong reduction in IFNγ release. These data obtained in hu-BLT mice [91] confirmed previously published findings in wild-type and Rag2^−/−^ mice, where the Zol treatment caused a reduced release of IFNγ in gingiva and an increased one in bone marrow [92]. Moreover, in PBMCs and other peripheral tissues, NK cells showed high cytotoxicity and IFNγ release after Zol treatment, and low cytotoxic activity and IFNγ secretion after Dmab treatment [91]. Thus, Dmab seems to be more suppressive than Zol for cells in peripheral tissues compared to ones in bone marrow. The suppression of IFNγ in gingiva inhibits immune cells and induces the deregulation of OB and OC activity (Figure 2) [93]. OCs may regulate NK cells [94,95], and the lack of IFNγ secretion by NK cells can affect stromal cells with a stem cell-like phenotype, which remain undifferentiated, making it easy for them to be killed by NK cells [96]. However, the cross-talk between NK cells and OCs in the bone microenvironment is largely unexplored, especially in cancer patients with bone metastases [97] who present an increased risk to develop MRONJ. Thus, future investigations perhaps performed directly on samples derived from cancer patients treated with ARD could help to better define the role of NK cells and OCs in the pathogenesis of MRONJ.

### 4.4. Macrophages

Macrophages are important immune cells that secrete a variety of inflammatory factors, activate T cells and play a critical role in both the early and late phases of wound repair [59]. In a rat model of MRONJ, macrophages proliferated and secreted at the level of the jaw bone included a large number of inflammatory cytokines, such as TNF-α, IL-1 and IL-6, which aggravated tissue damage [33]. Macrophages are categorized in different subsets; particularly, M1 and M2 are important for the process of wound healing, intervening at different times. Indeed, M1 macrophages are pro-inflammatory, acting in an early phase of the healing, while M2 are anti-inflammatory, being mainly active in the late phase of the healing process [98]. Therefore, the M1/M2 ratio determines the normal healing of an injured tissue. BPs interfere with macrophage survival and differentiation in the mucosa of MRONJ patients. When the M1/M2 ratio is significantly higher than normal, there is a dysregulation of macrophage activity [55,99]. In a recent clinical study, patients in the early stage of MRONJ showed a high density of M2 macrophages in mucosal tissues bordering the necrotic bone, whereas in advanced stages there were more M1 macrophages [100]. Some studies focused on the explanation of this M1 polarization, showing that it can be regulated by different mechanisms such as the increased level of IL-17, the activation of the Toll-like receptor 4 (TLR-4)-mediated nuclear factor kappa B (TLR-4/NF-κB) signaling pathway and the upregulation of the expression of histone demethylase [22,55,101].

The literature data show that Dmab does not seem to interfere with macrophage viability and differentiation [102], but more recently, an interesting murine model of chemotherapy/denosumab-induced ONJ demonstrated that Dmab discontinuation for 2 weeks maintained the M1 macrophages and promoted the accumulation of M2 ones in the connective tissue of tooth extraction sockets, restoring the correct M1/M2 ratio and improving the healing of MRONJ-like lesions [103].

In another model, both N-BPs and Dmab were compared to identify potential different mechanisms of action on macrophage subsets in MRONJ. The authors reported that, in the early stages of MRONJ, combination therapy with cyclophosphamide (CY)/Dmab did not affect macrophages expressing lymphatic endothelial markers, which are able to transdifferentiate into lymphatic endothelial cells and lymphangiogenesis, while CY/Zol suppressed the transdifferentiation of macrophages and lymphangiogenesis [74]. Furthermore, CY/Dmab showed a stronger anti-angiogenic capability in the soft tissue of the tooth extraction socket compared to that of CY/Zol due to the increase in vascular endothelial growth factor A (VEGF-A) [104], while CY alone did not impair, but only delayed wound healing in tooth extraction sockets [105].

These data confirm the important role of macrophages in the pathogenesis of MRONJ and suggest different mechanisms of action of N-BPs and Dmab. For a deep revision on the role of macrophages in MRONJ, we suggest reading the recent review by Gkouveris et al. [59].

### 4.5. Dendritic Cells

DCs are a central player in the immune system since they are antigen-presenting cells that activate the adaptive immune response [106,107]. BPs have been demonstrated to inhibit DC maturation and activation, impairing phagocytosis and potentially promoting immunosuppression or infectious complications [108,109]. Furthermore, Zol has been found to hinder DC [110] cell-dependent activation [57]. DCs contribute to the maintenance of the integrity of oral mucosa and alveolar bone. Indeed, the rate of MRONJ after dental extraction was found to be higher in DC-deficient mice in [20]. In the oral cavity of mice treated with Zol, DC functions were altered and the bacterial load was increased, leading to impaired bone regeneration after extraction. In addition, N-BPs increased the allostimulatory activity of DCs on naive T cells, which produce IFNγ [108] and are able to upregulate the expression of β-defensin-3, explaining the reason why β-defensin 3 is high in MRONJ samples [111].

There is a lack of data on the effect of Dmab on DCs within the context of ONJ, but it is known that the maintenance, homeostasis, maturation and activation of DCs during an immune response are sustained by molecules of the TNF receptor superfamily, such as RANK and CD40, that are strongly expressed on DCs. RANKL stimulates DC survival, as the RANKL–RANK interaction exerts a pro-inflammatory stimulus which could selectively either promote or suppress immunity as determined by the specific phase of the immunity cycle at which this pathway is activated [112]. Thus, it is reasonable to think that Dmab may exert an effect on DCs, which deserves investigation.

### 4.6. Neutrophils

Neutrophils are essential for correct wound healing, and in vitro studies have reported that N-BPs reduce chemotaxis, phagocytosis and the oxidative burst of neutrophils, compromising normal wound healing; thus, neutrophils also play a role in the pathogenesis of N-BP-associated ONJ [58]. N-BPs reduce the life span of neutrophils, but not their differentiation ability [113]. Neutrophil infiltration was detected in the bone of both MRONJ patients [114] and in a mice model [110], and they appear to juxtapose apoptotic OCs [115]. Data derived from animal studies showed that N-BPs increased the neutrophil numbers and pro-inflammatory factors, such as TNFα, IL-1β, inducible nitric oxide synthase (iNOS) and NF-kβ, at MRONJ sites [116].

## 5. Conclusions

The role of the immune system in the pathogenesis of MRONJ is becoming more evident since ARD can deeply modulate different immune subsets, which, in turn, regulate bone cells and inflammatory conditions. In MRONJ, as well as in other bone diseases, osteoimmunology represents a key to understand pathogenic mechanisms and to identify potential new therapeutic targets. The anti-RANKL antibody, Dmab, is a typical example of a drug specific for a target, as it acts on both bone tissue and on the immune system, by reducing OC differentiation and modulating the cellular immune subsets, respectively. Unfortunately, compared to the vast literature available on N-BPs, only scarce evidence can be easily retrieved regarding Dmab in animal models. This drug recognizes only human and monkey’s RANKL, and thus the studies conducted on murine models use recombinant RANKL inhibitors, such as Rank-FC, which is only a Dmab analogous. It is likely that this is one of the major reasons why data on the effects of Dmab on some immune subsets are lacking. Nonetheless, further studies on biological samples derived from treated patients would help to explain how Dmab affects T-cell subsets and their interactions with bone cells. These data are relevant, since we think that an accurate analysis of patients’ immune systems could help physicians to immediately identify subjects at a high risk for ONJ.

## Data Availability

The data are contained within the article.
